# Comparison between high-velocity low-amplitude manipulation and muscle energy technique on pain and trunk neuromuscular postural control in male workers with chronic low back pain: A randomised crossover trial

**DOI:** 10.4102/sajp.v76i1.1420

**Published:** 2020-10-26

**Authors:** Leandro A. Sturion, Alexandre H. Nowotny, Fabrice Barillec, Gilles Barette, Gabriela K. Santos, Fellipe A. Teixeira, Karen P. Fernandes, Rubens da Silva

**Affiliations:** 1 Programs in Rehabilitation Sciences UEL/UNOPAR, LAFUP-UNOPAR, Faculty of Physical therapy, Universidade Pitagoras, Londrina, Brazil; 2 Département des Thérapie Manuelle, Gilles Barette Formations, Saint Cyr du Ronceray, Valorbiquet, France; 3 Département des Cadre de santé-kinésithérapeute, Thérapie Manuelle, Gilles Barette Formations, Valorbiquet, France; 4 Escuela de Osteopatia de Madrid, Parque Taquaral, Campinas, Brazil; 5 Département des sciences de la santé, Lab BioNR, CUpht, Université du Québec à Chicoutimi (UQAC), Saguenay, Québec, Canada

**Keywords:** low back pain, osteopathic manipulative treatment, high-velocity low-amplitude, muscle energy, electromyography, postural balance, physiotherapy, biomechanics

## Abstract

**Background:**

A therapeutic recommendation for restoring function in individuals with chronic low back pain (CLBP) is manual therapy through manipulative spinal or muscle energy techniques.

**Objectives:**

To compare the effectiveness of two osteopathic manipulative techniques on clinical low back symptoms and trunk neuromuscular postural control in male workers with CLBP.

**Method:**

Ten male workers with CLBP were randomly allocated to two groups: high-velocity low-amplitude (HVLA) manipulation or muscle energy techniques (MET). Each group received one therapy per week for both techniques during 7 weeks of treatment. Pain and function were measured by using the Numeric Pain-Rating Scale, the McGill Pain Questionnaire and the Roland Morris Disability Questionnaire. The lumbar flexibility was assessed by Modified Schober Test. Electromyography (EMG) and force platform measurements were used for evaluation of trunk muscular activation and postural balance, respectively at three different times: baseline, post intervention, and 15 days later.

**Results:**

Both techniques were effective (*p* < 0.01) in reducing pain with large clinical differences (-1.8 to -2.8) across immediate and after 15 days. However, no significant effect between groups and times was found for other variables, namely neuromuscular activation and postural balance measures.

**Conclusion:**

Both techniques (HVLA thrust manipulation and MET) were effective in reducing back pain immediately and 15 days later. Neither technique changed the trunk neuromuscular activation patterns nor postural balance in male workers with LBP.

**Clinical implications:**

These results may facilitate clinical decision-making for CLBP management in physiotherapy programs.

## Introduction

Chronic low back pain (CLBP) is a very common condition and one of the most important public health problems in the world. Chronic low back pain prevalence can reach 70% of the population, especially in the economically active age (Majid & Truumees 2008). Chronic low back pain can further lead to various socio-economic problems such as long-term disability and absence from work, which, in turn, increases the absenteeism of adult workers (Wolter et al. 2011; Yang et al. 2016). Amongst workers with CLBP, the prevalence can reach 27% in women and 24% in men (Yang et al. 2016). Some evidence shows, however, that the prevalence of back pain in male workers, between 35 and 55 years old, can reach 28% (Yang et al. 2016).

Chronic low back pain may be associated with impaired motor control and increased postural instability (Shigaki et al. 2017). Stabilising muscle function and coordination are often impaired in individuals with CLBP (Panjabi 2006; Shigaki et al. 2017). Decreased back endurance has been shown to be a predictor of first-time CLBP occurrence (Biering-Sorensen 1984) and of long-term back-related disability (Enthoven et al. 2003). Trunk muscle fatigue can increase neuromuscular deficits, resulting in brief uncontrolled intervertebral movements, lumbar spine instability and back pain (Brumagne et al. 2008; Granata & Gottipati 2008; Johanson et al. 2011; Panjabi 2006). In addition, poor lumbar proprioception has been reported in some individuals with CLBP (Brumagne et al. 2008; Brumagne, Cordo & Verschueren 2004). Balance performance is also decreased in individuals with CLBP during bipedal standing and one-legged stance (Da Silva et al. 2016; Lafond et al. 2009; Shigaki et al. 2017). In fact, in a recently published study we found that participants with CLBP presented significantly poorer balance during a one-legged stance, as measured by centre of pressure (COP) variables, than participants without CLBP (effect size of *d* = 1.44 for younger adults and *d* = 0.40 for older individuals; Da Silva et al. 2016). Some theories (biomechanical model, pain adaptation model and reflex spasm pain model) based on the interpretation of changes in trunk muscle activation may help to better explain these negative results on postural control measures in individuals with CLBP (Van Dieen, Selen & Cholewicki 2003). People with CLBP have been shown to have different trunk activation patterns depending on the task (hyper- or hypoactive) compared with those without CLBP (Van Dieen et al. 2003). A recent study (Da Silva et al. 2019) reports further that individuals with CLBP have lower trunk activation during balance performance and increased co-activation to maintain the task, which further supports these hypotheses for neurophysiological mechanism-associated back pain (Panjabi 2006). This evidence is supported by the use of high-tech instruments such as force platforms and electromyography (EMG) measures, which can precisely quantify trunk neuromuscular activation patterns and postural stability.

A therapeutic indication for restoring function in individuals with CLBP is manual physiotherapy through manipulative spinal therapy, which is recommended by international guidelines as a non-drug intervention in the management of nonspecific low back pain (LBP; Koes et al. 2010). In some countries this therapy is considered a first treatment option, whilst in others it is recommended as an essential treatment (Qaseem et al. 2017) component associated with exercise (NICE 2016). More recently, the use of manipulative treatment techniques performed by professionals working in the osteopathy area has been suggested for some cases, aiming to improve pain and function (Delitto et al. 2012).

Some evidence has demonstrated the efficacy of osteopathic manipulative treatment (OMT) in LBP (Hamilton, Boswell & Fryer 2007; Licciardone, Brimhall & King 2005; Rubinstein et al. 2012; Wilson et al. 2003). However, there is little scientific evidence about the physiological and neuromuscular effects of these treatment techniques in individuals with CLBP, especially in workers, which is the focus of our study.

Two manipulative osteopathic techniques have been suggested: the high-velocity low-amplitude (HVLA) thrust manipulation (Hamilton et al. 2007; Rubinstein et al. 2012) and the muscle energy technique (MET; Franke et al. 2015). Both aim mainly to restore mobility and function and are used for the reduction of pain (Hamilton et al. 2007). High-velocity low-amplitude thrust manipulation is a passive technique, applied near the end of the joint range of motion (ROM) and can cause cavitation (Evans 2002; Unsworth, Dowson & Wright 1971). There are two hypotheses often cited to explain the decrease in pain with this technique: (1) joint manipulation activates mechanoreceptors that inhibit nociceptive afferents (gate control theory; Hamilton et al. 2007; Melzack & Wall 1965), and (2) manipulation releases adhesions in the joint, reduces zygapophyseal peri-articular oedema (improving the drainage) and consequently improves the ROM (Hamilton et al. 2007; Harvey & Descarreaux 2013). On the contrary, MET is an active or passive technique, characterised by voluntary contractions and relaxations of the patient’s muscles (Chaitow 2006; Franke et al. 2015; Hamilton et al. 2007; Rubinstein et al. 2012) and uses reciprocal inhibition physiological mechanisms; a muscle contraction inhibits or decreases the motor neurons’ excitability that innervates the antagonist muscle (Chaitow 2006). This technique can be used to mobilise restricted articulations and reduce pain and disability (Chaitow 2006; Hamilton et al. 2007; Wilson et al. 2003). Few studies have investigated and compared the effect of both HVLA thrust manipulation and MET on pain, disability and trunk neuromuscular measures, namely postural control and muscular activation in workers with CLBP. The main purpose of our study was thus to compare the effect of HVLA thrust manipulation and MET on the clinical symptoms and neuromuscular and postural control of the trunk in adult male workers with CLBP. The hypotheses are that both techniques would be beneficial in reducing the clinical symptoms and change the mobility, postural control and trunk neuromuscular function of individuals with CLBP based on the hypothesis presented previously and supported by the literature (Chaitow 2006; Wilson et al. 2003).

## Method

Our crossover clinical trial was performed at the Laboratory of Functional Evaluation and Human Motor Performance (LAFUP-UNOPAR) and Physical Therapy Clinic at the Universidade Pitágoras UNOPAR.

### Sample

**FIGURE 1:**
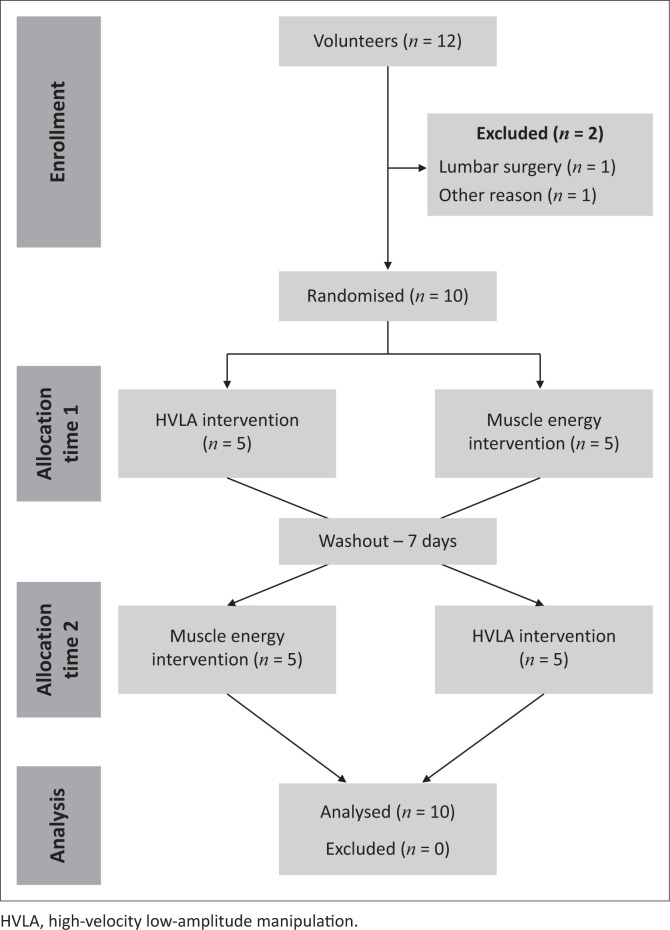
Flowchart – Consolidated Standards of Reporting Trials (CONSORT).

A convenience cohort of 12 volunteers was recruited from a local community of active workers, aged between 35 and 55 years. Figure 1 shows the recruitment and design flowchart of our study.

The inclusion criteria were as follows: a history of lumbar or lumbosacral pain, without proximal radicular pain; presence of chronic pain defined as daily or almost daily pain for a minimum of 3 months (Da Silva et al. 2016, 2019; Shigaki et al. 2017); current lumbar pain of unknown mechanical origin (muscular or passive structures); not participating in rehabilitation programmes; and not having practised regular physical activity in the last 3 months. The exclusion criteria were as follows: presenting ‘red flag’ signs to manual therapy (e.g. tumours, osteoporosis and so on); having a history of lumbar or locomotor surgery; presenting any type of neurological, cardiorespiratory and/or orthopaedic disease of high severity; and presenting psychiatric disorder and/or attention and speech disorders (Hamilton et al. 2007; Licciardone et al. 2013; Rabin et al. 2014).

### Power analysis

The sample size calculation was based on the outcome measure of self-reported pain from a previous randomised crossover trial reporting the efficacy of OMT for CLBP management (Rabin et al. 2014). This study used similar inclusion and exclusion criteria, outcomes in assessment (Numeric Pain-Rating Scale [NPRS]: pain; Fear-Avoidance Beliefs Questionnaire [FABQ]: fear and beliefs; lumbar spine mobility) and intervention (treatment sessions over a period). A power analysis was performed, and a sample of 10 participants was determined to detect a 12-mm change on a 100-mm visual analogue scale (VAS) for pain as an immediate effect, assuming a power of 80% and an *α* value of 0.05.

### Randomisation and blinding

An evaluator not involved in our study was responsible for group randomisation (i.e. HVLA group; MET group), which was generated by using *random.org* and distributed in sealed and opaque envelopes to the therapist performing the interventions (Figure 1).

Owing to the nature of our study, only the authors responsible for the main outcome measures were blinded to the intervention allocation.

### Instrumentation and measurements

To collect demographic data, symptomatic characteristics of individuals’ CLBP and their work activities, all participants were asked to answer clinical questionnaires. Following Langevin et al. (2015), our study divided the type of labour activity into three categories: sitting; standing and sitting; carrying and holding a load.

### Trunk neuromuscular activation pattern (electromyography)

**FIGURE 2:**
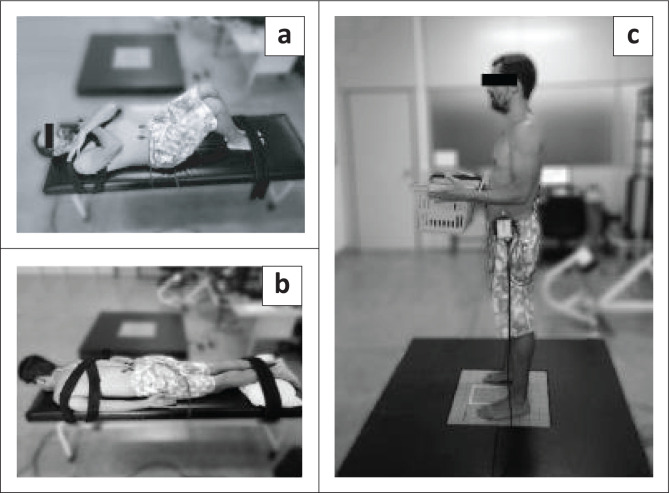
Positioning and stabilisation during the abdominal (rectus abdominis) (a) and paravertebral (multifidus-L5 and iliocostalis-L3), (b) neuromuscular activity measuring and (c) bipodal support with load.

The trunk neuromuscular activation patterns were evaluated by using an EMG. The EMG signal was captured with six pre-amplified (gain: 1000) active surface electrodes, by using Bagnoli-8 EMG system (Delsys Inc., Wellesley, MA, USA). After trichotomy and skin cleaning, the electrodes were positioned bilaterally on the target trunk muscles (Figure 2a and 2b) generally used for different postures and balance performance (Da Silva et al. 2019; Lafond et al. 2009; Van Dieen et al. 2003) namely: rectus abdominis (RABD), iliocostalis (ILC-L3) and multifidus L5 (MU-L5) levels, following the Surface EMG Guide for Non-Invasive Assessment of Muscles (SENIAM 1999) and the protocol used by Larivière et al. (2011) and Da Silva et al. (2005; 2009; 2019). The ground electrode was placed on C7 (Larivière et al. 2011). For EMG normalisation and to determine the percentage of muscle activity during tests, the participants first performed two 5-s maximal voluntary contractions (MVC) with 1-min interval between contractions (Da Silva et al. 2009). Participants contracted the RABD muscle in supine with knees flexed (Figure 2a), and the paravertebral muscles in prone (MU-L5 and ILC-L3; Figure 2b). An MVC protocol was adapted from Da Silva et al. (2005). During two 5-s MVC, a signal amplitude analysis (peak in root mean square [RMS_MVC_]) was calculated for normalisation purposes (Da Silva et al. 2009). The neuromuscular activities collected during the tasks for the normalisation procedure, as supported by Da Silva et al. (2009; 2019), from 60-s bipodal support with and without external load, were computed to obtain the average EMG value computed from 250 ms RMS time-window to finally reached (RMS_MEAN-TASK_). The neuromuscular activities of the trunk across the tasks were normalised in the equation as follows (Da Silva et al. 2009):

Equation: %RMS (muscle activation level) = [(RMS_MEAN-TASK_/RMS_MVC_) × 100%]

All EMG data processing was performed by using Delsys system *EMG work analysis program* (Version 4.0; Delsys, MA, USA) and utilised MATLAB sub-routines (Version 8.0; The MathWorks Inc., Natick, MA, USA, release 14).

### Static postural balance (force platform)

A BIOMEC 400 force platform (EMG System do Brasil, SP Ltda.) was used for evaluation of the static postural balance (Figure 2c), with and without an external load on the trunk (Shigaki et al. 2017). All force signals recorded by the platform were collected at a 100 Hz sampling frequency. The Bioanalysis software of the BIOMEC 400 platform, compiled with MATLAB (The Mathworks, Natick, MA) analysis routines, acquired and managed the equilibrium parameters in the two experimental conditions (with and without load). The main equilibrium parameters were based on the COP and consisted of the ellipse area 95% COP displacement (A-COP cm^2^) and the mean velocity (VEL cm/s) of the COP oscillations in the direction of the movement: anteroposterior (A/P) and mid-lateral (M/L; Da Silva et al. 2013; Shigaki et al. 2017). After familiarisation, the balance tasks (bipodal support with and without load) were performed and lasted for 60 seconds with a 3 min rest between trials. The balance protocol for the task without load consisted of the participant standing on the force plate with his feet parallel; eyes were open and fixed on a target positioned at eye level and 2 m ahead; and arms outstretched at the side of the trunk (Shigaki et al. 2017). For the task with load, the same positioning was used except that the shoulders were placed in a neutral position and the elbows semi-flexed while holding a box weighing 10% body mass close to the anterior trunk (Shigaki et al. 2017; Figure 2c). Before and after the task with load a participant reported his subjective fatigue using the CR-10 Borg scale proposed by Dedering et al. (2000). An external trigger (EMG System do Brasil, SP Ltda.) was used to simultaneously collect COP measures from the force platform signals and EMG measurements of trunk neuromuscular activity.

### Clinical symptoms

The following validated questionnaires assessed participants’ clinical symptoms: NPRS and McGill Pain Questionnaire - Short Form (SF-MPQ) assessed the pain level. The NPRS has an 11-point scale with a score ranging from 0 (no pain) to 10 (worst pain imaginable) (intraclass correlation coefficient [ICC] = 0.94 [95% CI: 0.90–0.96]) (Costa et al. 2011). The SF-MPQ presents 15 descriptors of pain sensation (11 sensorial, 4 affective) with 4 possible gradations (0 – none to 3 – severe); the sum of the responses varies from 0 to 45 (corresponds to the worst pain sensation) (ICC = 0.96 [95% CI: 0.94–0.98]; Costa et al. 2011).

The Roland Morris Disability Questionnaire (RMDQ) Brazilian version (ICC = 0.94; Nusbaum et al. 2001) assessed the functional aspects. The RMDQ presents 24 statements that evaluate disability as a result of LBP. The score ranges from 0 (no disability) to 24 (severe disability).

The FABQ-Brazil (Abreu et al. 2008) assessed the beliefs and fears. This instrument contains two subscales: physical activities (FABQp) (ICC = 0.84) ranging from 0 to 24; and work activities (FABQw) (ICC = 0.91) ranging from 0 to 42 – high score reflects greater fear influence.

Finally, all the participants performed the Modified–Modified Schober Test (here just called Schober) to measure lumbar spine flexion mobility (Cidem, Karacan & Uludag 2012). The evaluators palpated the inferior margins of the posterior superior iliac spines and marked the intersection of them by drawing a horizontal line (first mark). A second mark was drawn 15 cm above the midpoint of the first mark. The participant was then asked to bend forward as far as possible (no knees flexion) until the onset of the pain. The new distance between the first and second marks was measured. This measurement expressed lumbar flexion mobility (Cidem et al. 2012; MacDermid et al. 2014). This test has been shown to be reliable (high to very high intra-trial reliability [ICCs 0.84–0.98]; moderate to high inter-rater reliability [ICCs 0.75–0.82] for LBP and non-LBP groups) in assessing the lumbar spine mobility (MacDermid et al. 2014).

### Intervention

**FIGURE 3:**
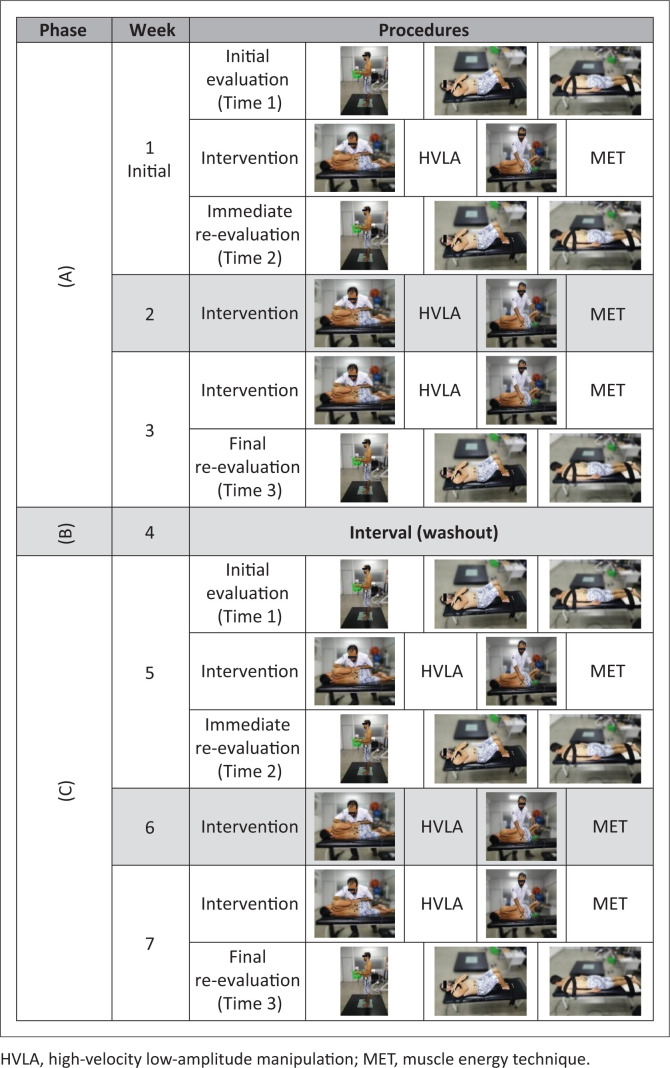
Crossover study interventional model description.

The crossover intervention protocol started directly after the completion of baseline measurements. Three different phases were applied for the intervention (Figure 3): (Phase A) 3 weeks of either HLVA or MET technique – intervention randomly across participants; (Phase B) a 1-week washout period for both (Martins et al. 2015; Rabin et al. 2014); (Phase C) 3 weeks of intervention for other technique that those of Phase A (Figure 3). Both HLVA and MET interventions were administered once per week. All measures were collected at the same time for the two treatment phases (i.e. phases A and C, Figure 3): before (Time 1) and immediately after (Time 2) the first treatment session, and after the third treatment session (Time 3), which occurred 15 days after Time 1. Participants were instructed and reminded (by call and messages) not to change their daily habits throughout our study.

### Specific vertebral level assessment

Two specialised physiotherapists, osteopaths, following the osteopathic model (Wilson et al. 2003), evaluated the lumbar spine (L1–L5) searching for dysfunction (low mobility). The dysfunction of vertebral segments received the correspondent technique application according to the respective intervention group. Each therapist applied one of techniques (HVLA or MET) throughout our study; thus, the same therapist evaluated and treated the same intervention group.

### Techniques application

**FIGURE 4:**
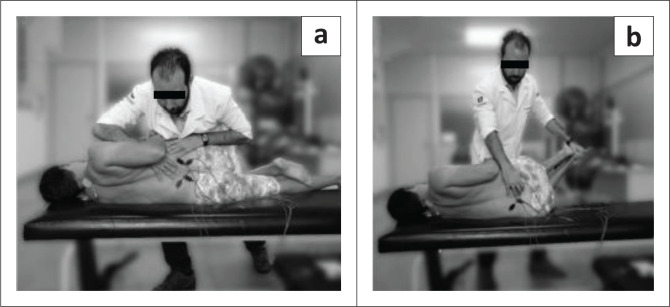
(a) High-velocity low-amplitude manipulation thrust manipulation positioning and (b) muscle energy technique positioning.

Before applying the techniques, the therapists informed participants about HVLA and MET application and demonstrated them. The patients were dressed as illustrated in Figure 4. One specialised therapist performed the HVLA thrust manipulation following these steps, patient in side lying knees flexed (restriction side up), the therapist: (1) flexed the hip until motion is detected at the target segment; (2) rotated the upper trunk backwards until motion is detected at the target segment; (3) rolls the patient towards him/her and stretches the segment to its end range; and (4) applies an HVLA thrust (as described by Rabin et al. 2014; Figure 4a). This technique was performed once per week for three weeks.

The other specialised therapist applied MET with the patient in side lying with knees bent (restriction side down), whilst the therapist: (1) palpated the target segment and extended the patient’s legs until motion was detected at the target segment; (2) flexed patient’s trunk superiorly until motion was detected at the target segment; (3) flexed trunk inferiorly until motion was detected at the target segment; (4) rotated patient’s trunk until motion was detected at the target segment; and (5) side-bent patient’s trunk until motion was detected at the target segment. Upon command, the patient pushed his/her legs down into examiner’s hand for a 5-s isometric contraction (as described by the Wilson et al. 2003 and Chaitow 2006; Figure 4b). This process was repeated three times for each of the once per week intervention.

### Statistical analysis

For the analysis of variance the Shapiro Wilk test supported by the Levene test confirmed the normal distribution of the sample data for all variables. Once normality was established, a multivariate analysis of variance (MANOVA) was used for each dependent variable to explore its influence on the groups (HVLA, MET), time of measurement (pre-intervention, immediately after 3 weeks of intervention and 15 days later) and the interaction between these factors. A Tukey’s post hoc test located the significant differences between the times pre-, post- and post-intervention 15 days. Our study analysed all dependent variables for both techniques and computed the detectable clinical differences between the pre- and post-intervention 15 days measures. The effect size (ES) was used to determine the magnitude of the changes and improvements. The statistical programme SPSS (version 20.0 for Windows) was used to perform all statistical analysis with an alpha level of 5% (*p* < 0.05).

### Ethical consideration

The study followed the Resolution 466/12 of the National Health Council. Ethical approval of our study was obtained from the Research Ethics Committee of the Universidade Pitagoras UNOPAR (#1.626.690, 06 July 2016). The clinical trial register was also approved (ClinicalTrials.gov – NCT02983435). The evaluators informed all participants about the purpose of our study as well as the experimental protocol. The participants in agreement with the study signed the free and informed consent form.

## Results

All participants completed whole the intervention and the procedures were well tolerated without any adverse events. The participants had a mean age of 44 years, mass = 81 kg and height = 1.73 m. Fifty per cent of the participants reported having back pain for 24–36 months; half of the participants worked almost exclusively in the sitting position. After the random allocation, the two groups were similar at baseline for clinical symptoms and the mobility test (*P* > 0.05).

Table 1 presents the HVLA and MET effects for pain across time (before, post intervention and after a further 15 days). Both techniques were significantly (*p* < 0.01) effective in reducing pain according to the numerical scale immediately after the first session (HVLA = 26% [mean percentage difference] of decrease vs. time 1; MET = 39%) and after 15 days of intervention (HVLA = 52%; MET = 73%). Table 1 presents the effect size for pain improvement after 15 days: clinical difference of 1.8 for the HVLA group (ES *d* = 0.78) and 2.8 for the MET group (ES *d* = 1.27). There were no statistically significant changes in the disability and fears and beliefs questionnaires values, but the data do indicate an improvement. The Schober’s test improved for both techniques after 15 days, but with weak/null effect sizes (HVLA group: ES *d* = 0.18 and MET group: ES *d* = 0.05).

**TABLE 1:** Effect of interventions (high-velocity low-amplitude thrust manipulation and muscle energy technique) on clinical variables from initial time (1 = pre-intervention); immediate (2 = post-immediate intervention on first day); and after 15 days (3 = post-final intervention).

Variables	Groups (*n* = 10)	Time of intervention			Clinical differences	Groups *p*	Times *p*	Interaction *p*
		Initial (1)	Immediate (2)	15 days (3)				
NPRS (pain)	HVLA	3.4 (2.3)	2.5 (3.1)	1.6 (1.8)	−1.8	0.816	< 0.01*	0.785
	MET	3.8 (2.2)	2.3 (1.8)	1.0 (1.3)	−2.8	-	Post hoc 1 ≠ 3	-
SF-MPQ	HVLA	8.2 (7.9)	-	7.2 (7.7)	−1.0	0.883	0.435	0.769
	MET	9.1 (4.4)	-	6.9 (4.5)	−2.2	-	-	-
RMDQ	HVLA	7.0 (5.5)	-	6.0 (5.0)	−1.0	0.714	0.513	> 0.99
	MET	6.4 (5.9)	-	5.4 (3.7)	−1.0	-	-	-
FABQp	HVLA	12.3 (8.0)	-	10.0 (8.1)	−2.3	0.293	0.444	0.852
	MET	14.4 (8.2)	-	13.0 (5.2)	−1.4	-	-	-
FABQw	HVLA	15.1 (11.2)	-	13.0 (9.1)	−2.1	0.579	0.625	0.845
	MET	12.8 (8.8)	-	11.9 (8.9)	−0.9	-	-	-
Schober (cm)	HVLA	5.4 (1.6)	-	5.7 (1.0)	+0.3	0.620	0.708	0.832
	MET	5.3 (1.9)	-	5.4 (1.4)	+0.1	-	-	-

FABQp, Fear-Avoidance Beliefs Questionnaire – physical activities; FABQw, Fear-Avoidance Beliefs Questionnaire – work activities; HVLA, high-velocity low-amplitude manipulation; MET, muscle energy technique; NPRS, Numeric Pain-Rating Scale; RMDQ, Roland Morris Disability Questionnaire; SF-MPQ, Short Form – McGill Pain Questionnaire.

Note: Mean values and standard deviation are given in parenthesis. Clinical difference is detectable. Negative values (NPRS, SF-MPQ, RMDQ, FABQp e FABQw) and positive values (Schober flexibility test) show clinical evolution with intervention.

* Significant difference across times from intervention by post hoc analysis of ANOVA (*p* < 0.05).

Table 2 (balance without external load) and Table 3 (balance with external load) present the results for trunk neuromuscular activation behaviour and postural balance parameters during bipodal support tasks. No significant differences between groups and times of measurements were observed for both %EMG and COP variables across the two tasks (Table 2 for no load and Table 3 with load). However, with regard to neuromuscular activation, both groups had detectable clinical differences in neuromuscular activation for the MU muscle (%MU-L5) in which there was an increase after 15 days for HVLA (mean = 2.1% increase with and without load) as well as for MET (mean = 1.2% increase with and without load) but the changes were not statistically significant.

**TABLE 2:** Changes in trunk neuromuscular activation and postural control during balance bipodal in standing task without external load.

Variables	Groups (*n* = 10)	Time of intervention			Clinical difference	Groups *p*	Times *p*	Interaction *p*
		Initial (1)	Immediate (2)	15 days (3)				
% MU-L5	HVLA	20.7 (12.9)	23.0 (15.9)	23.3 (14.8)	+2.6	0.391	0.932	0.956
	MET	25.1 (12.1)	25.7 (12.0)	25.2 (12.9)	+0.1	-	-	-
% ILC-L3	HVLA	24.2 (14.7)	25.2 (14.6)	21.0 (11.0)	−3.2	0.354	0.463	0.993
	MET	21.8 (8.9)	22.3 (11.6)	17.8 (7.4)	−4.0	-	-	-
% RABD	HVLA	9.3 (6.2)	10.3 (7.0)	10.3 (5.4)	+1.0	0.236	0.899	0.924
	MET	11.6 (4.9)	12.2 (3.7)	11.3 (5.1)	−0.3	-	-	-
% CO-ATIV	HVLA	54.4 (31.2)	50.9 (34.8)	60.8 (36.8)	+6.4	0.447	0.838	0.861
	MET	51.4 (19.8)	48.3 (19.9)	49.2 (25.9)	−2.2	-	-	-
A-COP (cm^2^)	HVLA	0.6 (0.1)	0.7 (0.1)	0.7 (0.2)	+0.1	0.223	0.723	0.330
	MET	0.8 (0.3)	0.8 (0.2)	0.6 (0.2)	−0.2	-	-	-
VEL A/P (cm/s)	HVLA	0.7 (0.09)	0.7 (0.08)	0.7 (0.07)	0.0	0.755	0.912	0.670
	MET	0.7 (0.1)	0.7 (0.08)	0.7 (0.1)	0.0	-	-	-
VEL M/L (cm/s)	HVLA	0.5 (0.06)	0.5 (0.08)	0.5 (0.07)	0.0	0.367	0.893	0.852
	MET	0.5 (0.04)	0.5 (0.06)	0.5 (0.04)	0.0	-	-	-

% MU-L5, percentage of activation of multifidus muscle at the L5; % ILC-L3, percentage of activation of ilicostalis at the L3; % RABD, percentage of activation of abdominal muscle; % CO-ATIV, percentage of activation from relationship between abdominal and multifidus muscles; A-COP, centre of pressure area sway; HVLA, high-velocity low-amplitude manipulation; MET, muscle energy technique; VEL A/P e M/L, velocity sway of COP in anteroposterior and mediolateral directions.

Note: Mean values and standard deviation are given in parenthesis. Clinical difference is detectable: positive values for increase in activation and negative values for decrease in activation and balance control.

**TABLE 3:** Changes in trunk neuromuscular activation and postural control during balance standing task with trunk external load.

Variables	Groups (*n* = 10)	Time of intervention			Clinical difference	Groups *p*	Times *p*	Interaction *p*
		Initial (1)	Immediate (2)	15 days (3)				
% MU-L5	HVLA	23.4 (14.7)	25.3 (15.1)	25.0 (16.1)	+1.6	0.136	0.888	0.998
	MET	28.9 (11.6)	31.0 (14.2)	31.1 (15.9)	+2.2	-	-	-
% ILC-L3	HVLA	25.5 (15.1)	27.0 (14.8)	22.0 (11.9)	−3.5	0.329	0.407	0.961
	MET	23.6 (9.8)	23.0 (10.7)	18.7 (7.6)	−4.9	-	-	-
% RABD	HVLA	10.6 (7.7)	11.4 (7.2)	10.8 (5.9)	+0.2	0.219	0.875	0.973
	MET	12.9 (3.6)	13.5 (5.2)	12.3 (5.8)	−0.6	-	-	-
% CO-ATIV	HVLA	71.4 (58.7)	68.7 (69.4)	67.2 (47.8)	−4.2	0.116	0.999	0.952
	MET	47.8 (26.1)	50.2 (24.7)	52.7 (26.5)	+4.9	-	-	-
A-COP (cm^2^)	HVLA	0.9 (0.4)	0.9 (0.4)	0.9 (0.4)	0.0	0.202	0.830	0.614
	MET	1.1 (0.4)	1.0 (0.4)	0.9 (0.4)	−0.2	-	-	-
VEL A/P (cm/s)	HVLA	0.8 (0.1)	0.8 (0.1)	0.8 (0.1)	0.0	0.655	0.694	0.499
	MET	0.8 (0.1)	0.9 (0.07)	0.8 (0.1)	0.0	-	-	-
VEL M/L (cm/s)	HVLA	0.5 (0.07)	0.5 (0.06)	0.5 (0.07)	0.0	0.546	0.789	0.951
	MET	0.5 (0.07)	0.5 (0.06)	0.5 (0.06)	0.0	-	-	-

% MU-L5, percentage of activation of multifidus muscle at the L5; % ILC-L3, percentage of activation of ilicostalis at the L3; % RABD, percentage of activation of abdominal muscle; % CO-ATIV, percentage of activation from relationship between abdominal and multifidus muscles; A-COP, centre of pressure area sway; HVLA, high-velocity low-amplitude manipulation; MET, muscle energy technique; VEL A/P e M/L, velocity sway of COP in anteroposterior and mediolateral directions.

Note: Mean values and standard deviation are given in parenthesis. Clinical difference is detectable: positive values for increase in activation and negative values for decrease in activation and balance control.

## Discussion

This study evaluated the effectiveness of two manual therapy techniques on clinical pain symptoms, postural control and trunk neuromuscular activation patterns in male workers with CLBP. Significant improvement was found only for pain in both techniques, with effect sizes of a 1.8 reduction for the HVLA group, and 2.8 for the MET group. No effect of the intervention was found for postural control and muscular activation variables, although non-significant clinical changes were observed (positive increase of trunk activation after intervention).

This is the first study to evaluate and compare these techniques in trunk neuromuscular activation patterns and postural control. Hamilton et al. (2007) evaluated these same techniques but only for reducing pain in the cervical region. Licciardone et al. (2005) also showed significant improvement for back pain after 12 weeks of osteopathic treatment. Other studies (Balthazard et al. 2012; Licciardone et al. 2013; Xia et al. 2016) used these techniques in combination with exercise and also demonstrated an immediate analgesic effect in patients with LBP. From these results and those of our study, these techniques appear to be similarly efficient in alleviating pain.

A systematic review and meta-analysis compared manipulation and mobilisation therapies for treatment of CLBP from 2018 (Coulter et al. 2018) and concluded that both therapies appear safe and are likely to reduce pain and improve specific function for patients with CLBP. In addition, manipulation produces a larger effect than mobilisation. Apart from pain intensity, our study failed to observe any effect on postural control, trunk activity responses and even in disability variables. However, Xia et al. (2016) found an improvement of pain level after therapy along with reduced disability and fear-avoidance beliefs amongst male and female participants in a sample of unemployed workers with LBP. These discrepancies between studies could be related to the fact that their patients had more severe clinical conditions at baseline (mean values) compared with our study: disability (RMDQ score = 9 in Xia vs. = 6.5 in our study), psychosomatic symptom (FABQ score = 14 in Xia vs. = 13 in our study) and pain (VAS = 55 mm in Xia et al. vs. = 3.5 in our study). These differences between the samples’ characteristics could explain, at least in part, the discrepancies of findings. Furthermore, Goertz et al. (2016) showed that vertebral manipulation (HVLA thrust manipulation) in the short term does not significantly alter postural balance responses, similar to our study. With regard to trunk neuromuscular activation, we cannot compare our results to the literature because this is the first study to investigate the effect of HVLA and MET on EMG activation patterns in trunk muscles. We expected a greater effect of MET on EMG activity because this technique aims at normalising hyper- or hypo-activity patterns through neurophysiological mechanisms recruited by repeated contractions or relaxations (Chaitow 2006; Franke et al. 2015). It must be remembered that MET is a technique characterised by active voluntary contractions and relaxations of the muscle associated with the passive movement of the therapist during application in patients (Chaitow 2006; Franke et al. 2015; Hamilton et al. 2007). Thus, there is a reciprocal inhibition physiological mechanism that could contribute to joint and muscular sprain relief and in turn improve the ROM (Chaitow 2006). Apparently, this technique could, in some way, mediate the activation of trunk muscles and reduce the pain. However, we were unable to demonstrate these results in our study.

The immediate results after the techniques’ application were almost equal to the initial values in both tasks (with and without load), that is the techniques did not create any neuromuscular changes. Thus, one can question recommendations given to patients (after the interventions) about avoiding some actions or activities. Both techniques showed non-significant improvements in lumbar spine flexion mobility. However, when we compared the ES after 15 days, we noticed that the HVLA group presented a result three times greater than the MET group, although the effect size is weak. We believe this small beneficial effect in the HVLA group is due to the fact that this technique is applied directly to the joints (Evans 2002; Hamilton et al. 2007; Rubinstein et al. 2012; Unsworth et al. 1971) and promotes a decrease in pain (gate control theory; Hamilton et al. 2007; Melzack & Wall 1965) and increased ROM (joint capsules stretch; Hamilton et al. 2007; Harvey & Descarreaux 2013). A study that evaluated the movement of the spine, but at angles, also showed improvement in mobility after intervention with manual techniques (Langevin et al. 2015), which could support our findings.

Finally, our study has some limitations. Only short-term effects were reported (i.e. management up to 15 days). The mobility evaluation was restricted to the sagittal plane only. No kinematic measures with a high-tech system were used, which limit our conclusions for this variable. The sample was small (although powered for our study) and only included men. Thus, more studies are necessary to determine further the implications of manual therapy on the long term response of clinical neuromuscular and biomechanical measures during a CLBP rehabilitation programme.

## Conclusion

Both techniques of manipulative treatment, HVLA thrust manipulation and muscle energy technique, are effective and comparable in reducing lumbar pain immediately and after a further 15 days post-intervention. However, the two techniques neither altered trunk neuromuscular activation patterns nor postural balance in standing with and without an external load on the trunk.
